# Application of Flotation Tailings as a Substitute for Cement in Concrete Structures for Environmental Protection and Sustainable Development—Part I: Sulfide Neutralization

**DOI:** 10.3390/ma18122804

**Published:** 2025-06-14

**Authors:** Vanja Đurđevac, Novica Staletović, Lidija Đurđevac Ignjatović, Violeta Jovanović, Nikola Vuković, Vesna Krstić

**Affiliations:** 1 Mining and Metallurgy Institute Bor, Alberta Ajnštajna 1, 19210 Bor, Serbia; lidija.ignjatovic@irmbor.co.rs; 2 Faculty of Ecology and Environmental Protection, University “Union-Nikola Tesla”, Cara Dušana 62-64, 11000 Belgrade, Serbia; nomstale@mts.rs; 3 Faculty of Management, Metropolitan University Belgrade, 11158 Belgrade, Serbia; violeta.jovanovic@metropolitan.ac.rs; 4 Institute for Technology of Nuclear and Other Mineral Raw Materials, Franchet d’Esperey 86, 11000 Belgrade, Serbia; n.vukovic@itnms.ac.rs; 5 Technical Faculty Bor, University of Belgrade, 19210 Bor, Serbia

**Keywords:** flotation tailings, sulfides, neutralization, hardened concrete, environmental protection, sustainable development

## Abstract

Flotation tailings (FT), as a product of the exploitation and processing of copper ore, represent a significant environmental and health risk due to the high content of heavy metals and sulfide compounds. Contemporary concepts of sustainable development and circular economy increasingly emphasize the need for rational use of resources and minimization of all types of waste, including mining waste. In this context, the reuse of flotation tailings in the construction industry represents a significant step towards closing the material flow in the mining and construction sectors. In order to reduce the negative impact of FT on the environment, the possibility of its application as a substitute for a portion of cement in the production of concrete was investigated. The main challenge is to reduce the negative impact of sulfides, originating from sulfide compounds, in order to achieve the desired concrete quality. Limestone aggregates of different size fractions (0/4, 4/8, 8/16 mm) were used for sulfide neutralization. Pyrite concentrate was used as a sulfide source, which together with FT provides the mixtures FT-7, FT-14, FT-25, and FT-40, with sulfur contents of 7.56, 13.84, 25.02, and 39.82%, respectively. FT mixtures were used as a substitute for Portland cement (PC) in the preparation of concrete. Test methods included XRD (X-ray diffraction), XRF (X-ray fluorescence), SEM (scanning electron microscopy), LP (leaching procedure), TCLP (toxicity characterization leaching procedure), assessment of acid eluate generation potential (AP—acid potential, NP—neutralization potential, and NNP—net neutralization potential), NEN (determination of heavy metals in cured concrete eluate), and UCS (uniaxial compressive strength of cured concrete). The results showed that the chemical characteristics of FT, as well as the chemical and mechanical properties of hardened concrete, allow the efficient use of these tailings in concrete mixes, which significantly utilizes FT, reduces the generation of mining waste, and contributes to the reduction of the negative impact on the environment and achieving sustainable development in mining.

## 1. Introduction

The increase in production in the mining industry contributes to the growth of gross national income, the creation of new jobs, and the improvement of the living standard of the population [1]. However, given the scale and nature of the industry, negative environmental impacts are significant, including land degradation and air and water pollution [2]. The exploitation of ores, both by surface and underground methods, requires the occupation of a large area of land for the storage of waste rock and flotation tailings (FT). FT, which is a by-product of the processing of metallic ores (such as iron, copper, and zinc ores), contains a high percentage of pyrite and toxic heavy metals, including lead, zinc, arsenic, and mercury, which can cause serious environmental pollution [3,4].

Exposure of FT to the atmosphere and surface waters leads to its leaching, resulting in the leaching of heavy metals and the formation of acidic drainage waters due to the oxidation of pyrite in the presence of water and oxygen. Thus, as Saedi et al. [5] emphasized, there is pollution of surface and underground water, as well as soil pollution. In addition, the flotation dam used for FT storage is a potential source of pollution if it is damaged or demolished [6]. For the production of one kilogram of copper from ore, usually 987 kg of FT is allocated, which represents a major risk to the environment and human health [7]. According to estimates published by Cacciuttolo and Cano [8], in 2022, the total amount of FT at the global level reached 14 billion metric tons.

Given these challenges, the question is whether it is possible to reuse FT for product development, providing economic benefits and reducing environmental risks.

Unfortunately, the extraction of metals and other valuable components from tailings does not solve the problem of waste disposal. In recent years, research has focused on the use of flotation tailings (FT) in cement paste backfill (CPB) for the purpose of filling abandoned underground mines [9,10]. Research on the application of tailings rich in the sulfide mineral pyrite in cement pastes was conducted by Martins and colleagues [11]. The studies were carried out using different cement matrices (Portland cement (PC), calcium aluminate cement (CAC), and calcium sulfoaluminate cement (CSA)). Cement was replaced with 30% tailings, and the testing period lasted 200 days. The results showed that tailings act as a stable mixture in PC, while in CSA, they form additional hydration products containing sulfates. Despite the high pyrite content in the hydrated pastes, no formation of expansive phases due to pyrite oxidation—which would affect paste durability—was observed. CSA cement demonstrated ecological compatibility, as it proved most suitable for immobilizing arsenic, zinc, cadmium, chromium, and copper.

Portland cement (PC), as the most commonly used ingredient of concrete, accounts for about 75% of the total price of concrete, but at the same time it has a significant negative impact on the environment due to CO_2_ emissions during its production, which accounts for approximately 5–7% of global CO_2_ emissions [12]. Therefore, replacing a part of the cement with FT could bring multiple benefits in terms of emissions reduction and environmental pollution. By using mining waste as a secondary resource in construction, value is created from waste, which is in accordance with the principles of circular economy and sustainable development [13,14]. This approach directly contributes to the principles of sustainable development by reducing the impact of the mining industry’s ecological footprint on the environment and promoting a circular economy, in accordance with the Sustainable Development Goals of the United Nations—especially goals 9 (Industry, innovation, and infrastructure), 11 (Sustainable cities and communities), 12 (Responsible consumption and production), and 13 (Climate action) [15].

Proper management and reuse of flotation tailings (FT), which contain heavy metals and sulfide minerals, would help ensure environmental protection. Using FT as a cement substitute in concrete production could address the release of heavy metals, the issue of tailings disposal, and the prevention of pollutant leaching into the environment [16]. Saedi and colleagues [5] used tailings with different sulfur contents—0.4% and 19%—as a partial cement replacement in proportions ranging from 20% to 80%. The results of the TCLP test showed that with increasing curing time, the leaching rate of toxic metals (cadmium, chromium, cobalt, lead, zinc, copper) decreased. The compressive strength of samples containing 20% tailings with 0.4% and 19% sulfur content increased over time, indicating that up to 20% of the cement can be replaced with tailings. However, the high sulfide content in tailings poses a problem, as it affects the formation of hydration products and leads to the creation of sulfide salts, which in turn weaken concrete quality.

Grinding FT results in finely ground materials that can be used as supplementary cementitious materials (SCMs). This fine material can act as an inert filler in concrete, providing more nucleation sites for hydration products [17]. Gutteridge and colleagues [18] found that inert fine materials exhibit a filler effect when replacing cement up to 20%. In the case of using fine sulfide tailings in mortar, low replacement ratios can improve mortar properties, even if the tailings have low pozzolanic properties or hydraulic reactivity [19]. These properties still require further investigation.

FT, as a by-product of the exploitation and processing of copper ores, represents a significant ecological challenge due to the presence of heavy metals and sulfide compounds. In order to reduce its negative impact on the environment, the possibility of using FT in combination with pyrite concentrate, as a substitute for part of the cement in the production of concrete, was investigated. Although the presence of sulfur from sulfide compounds can cause problems in the quality of concrete, the use of limestone aggregates allows the neutralization of these negative effects, thereby contributing to sustainable development through the use of mining waste in the construction industry.

## 2. Theory

Pyrite oxidation, as a key process in the creation of acid mine drainage (AMD), is a complex biogeochemical phenomenon that includes numerous reactions, both abiotic and biotic, with the role of various factors such as pH, temperature, presence of microbial catalysts, and oxygen concentration. The process mainly takes place in three phases: initial oxidation of pyrite by oxygen; oxidation of ferrous ions (Fe^2^⁺) to ferric ions (Fe^3^⁺); and in the final phase, when Fe^3^⁺ becomes the dominant oxidizing agent for pyrite.

### 2.1. Theoretical Basis of Pyrite Oxidation

Oxidation of pyrite in tailings, due to exposure to atmospheric influences, is a process through which pyrite oxidizes in the presence of oxygen and water. This biogeochemical process generates acidic solutions that affect the formation of AMD, the precipitation of soluble iron minerals, and the formation of acidic sulfate soils in the environment. Pyrite oxidation has a negative impact on the environment because it contributes to the mobilization of heavy metals and sulfates in natural watercourses, which has long-term effects on environmental pollution. This process is described in detail by Nordstrom [20].

I reaction: In the initial phase of oxidation, in a neutral and slightly basic environment, the oxidizing agent is oxygen. The oxidation of sulfur to sulfate leads to a decrease in pH, which can drop to as low as 4.5, shown by Reaction (1) [21]:

(1) $${2FeS}_{2\left(S\right)}+{7O}_{2(g)}+2H_{2}O\to 2{Fe}_{\left(aq\right)}^{2+}+{4SO}_{4(aq)}^{2-}+{4H}_{\left(aq\right)}^{+}$$

II reaction: The oxidation reaction of Fe^2^⁺ to Fe^3^⁺ is slow and determines the rate of the pyrite oxidation process, Reaction (2). The concentration of dissolved Fe^3+^ ions decreases due to the precipitation of Fe(OH)_3_. Bacteria such as *Thiobacillus ferrooxidans* can accelerate this reaction by increasing the rate of oxidation up to 6 times [22]:

(2) $${4Fe}_{\left(aq\right)}^{2+}+O_{2(g)}+{4H}_{\left(aq\right)}^{+}\to 4{Fe}_{\left(aq\right)}^{3+}+2H_{2}O_{\left(aq\right)}$$

III reaction: In highly acidic conditions (pH < 3), Fe^3^⁺ ions become the main oxidizing agent for pyrite [22,23]. Reaction (3) [24] shows how Fe^3^⁺ rapidly oxidizes pyrite in the presence of water and at low pH, which is crucial for the generation of acid mine drainage.

(3) $${FeS}_{2(S)}+{14Fe}_{\left(aq\right)}^{3+}+{8H}_{2}O_{\left(aq\right)}\to {15Fe}_{\left(aq\right)}^{2+}+{2SO}_{4(aq)}^{2-}+{16H}_{\left(aq\right)}^{+}$$

If the pH is >4.5, hydrolysis and precipitation of Fe^3+^ as Fe(OH)_3_ occurs, reducing the amount of oxidizing agent in the solution decreases, Reaction (4):

(4) $${Fe}_{\left(aq\right)}^{3+}+{3H}_{2}O_{\left(aq\right)}\to {Fe(OH)}_{3\left(s\right)}+{3H}_{\left(aq\right)}^{+}$$

If a neutralizing mineral, such as calcite (CaCO_3_), is present in the system where the pyrite oxidation process takes place, the pH increases, and the rate of oxidation decreases. Reaction (5) shows the dissolution of calcite, which neutralizes the acidity:

(5) $${CaCO}_{3(s)}+{2H}_{\left(aq\right)}^{+}\to {Ca}_{\left(aq\right)}^{2+}+H_{2}O_{\left(aq\right)}+{CO}_{2(g)}$$

**Figure 1:**
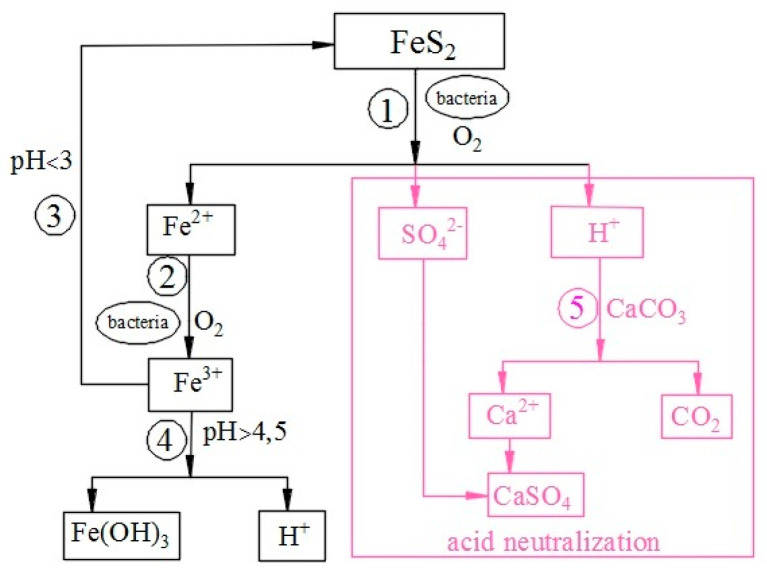
Schematic representation of pyrite oxidation and acid neutralization by calcite. The numbers from (1) to (5) represent the corresponding reactions (1) through (5).

Figure 1 shows the scheme of pyrite oxidation and acid neutralization, resulting from the reaction between pyrite and calcite. In the form of the algorithm in Figure 1, the reactions from (1) to (5) are presented.

**Figure 2:**
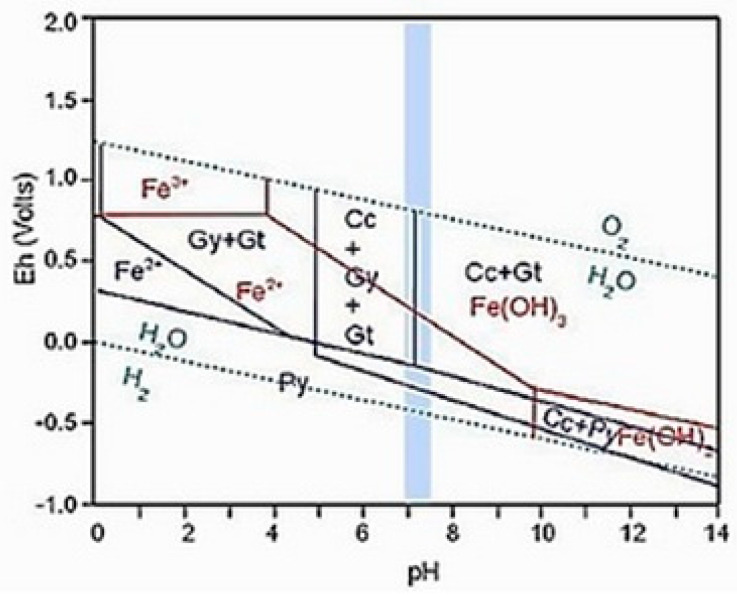
Eh—pH diagrams of C—Ca—Fe—S-—H_2_O (blue) and Fe-—O_2_-—H_2_O (red) systems at standard conditions (calculated using HSC Chemistry). Vertical blue bar: measured stable pH range of the seepage water [25].

Figure 2 shows the Eh-pH diagrams of the systems C-Ca-Fe-S-H_2_O (blue color) and Fe-O_2_-H_2_O (red color) at standard conditions, calculated with HSC Chemistry software [25]. The vertical blue band indicates the measured stable pH range of the seepage water, which is key to understanding the chemical reactions and stability of sulfide compounds in the system. These diagrams are important for the analysis of sulfide neutralization in flotation tailings, which are used as a substitute for cement in concrete structures. The presented data, shown in Figure 2, contribute to a better understanding of the process of neutralization and stabilization of sulfides, which enables a more efficient application of FT in concrete structures while reducing the negative impact on the environment and promoting sustainable development.

The key reaction, which defines the rate of pyrite oxidation and the formation of AMD, is the oxidation of Fe^2+^ in the presence of oxygen, as shown by Reaction (2). This reaction belongs to a group of very slow reactions under abiotic conditions. However, when occurring under biotic conditions in the presence of bacteria, it proceeds much faster. Because of this phenomenon, it was concluded that certain bacteria can act as catalysts for the Fe^2+^ ion oxidation reaction. There is a wide range of bacteria that can contribute to the biogeochemical oxidation of Fe^2+^ ions in the natural environment. This group of bacteria includes *Thiobacillus*, which are acidophilic and require pH < 3 for optimal growth and have been found in sulfide mine tailings [26,27]. Three species of *Thiobacillus* have been isolated from acidic mine waters [24]: *Thiobacillus ferrooxidans, Thiobacillus thiooxidans*, and *Thiobacillus acidophilus.*

### 2.2. Mechanism of Oxidation of Fe^2+^ Ions by Bacteria

*Thiobacillus* bacteria obtain energy by oxidizing reduced sulfur compounds. *Thiobacillus ferrooxidans* bacteria use iron (Fe^2^⁺) as an electron donor for their metabolic activity. The process of oxidizing Fe^2^⁺ to Fe^3^⁺ allows bacteria to obtain the energy needed for growth and the production of biologically important molecules, such as ATP and NADPH [28]. The efficiency of this process in bacteria is limited, as it has been found that only 3.2 to 30% of the energy produced by the oxidation of Fe^2^⁺ can be used for bacterial growth [29,30]. These bacteria are not only important for the oxidation of pyrite but also for sulfides and elemental sulfur.

Chemiosmosis, a process in which the energy from the oxidation of Fe^2^⁺ is used to transport protons (H⁺) across the cell membrane, explains the link between ATP production and Fe^2^⁺ oxidation [31,32,33].

### 2.3. Prevention and Remediation of Acidic Mine Drainage Waters

Acid mine drainage (AMD) is created in the vicinity of abandoned mines and tailings by the oxidation of mine waste, which is rich in sulfide minerals. Oxidation of pyrite, a sulfide mineral, in the presence of oxygen, bacteria, and water produces sulfuric acid and metal. The resulting oxidation products are responsible for the formation of low pH, high concentrations of sulfide ions, and heavy metals in AMD. AMD has a negative impact on the environment, as it can cause pollution of river watercourses and soil.

In active mines, the prevention of AMD is carried out by filling tailings, diverting water and making limestone trenches around the mine [34]. To solve the AMD problem, it is necessary to control the oxidation of Fe^2+^ ions, Reaction (2), which determines the rate of pyrite oxidation. Two ways of controlling the reaction have been proposed: thermodynamic control and kinetic control [35]. Two remedial systems can be used in vulnerable places where AMD has formed: active or passive AMD treatments [36].

## 3. Methods and Materials

### 3.1. Methods

XRF analysis was performed on a Rigaku Supermini 200 device and is a semi-quantitative analysis. The elemental analyzer Leco CS844 Carbon/Sulfur Determinator (St. Joseph, MI, USA) was used to determine the total sulfur in the FT. To determine the mineralogical composition of the FT and the concrete composite, an XRD analysis was used, performed with a RigakuMiniFlex 600 instrument with a D/teXUltra 250 high-speed detector and an X-ray tube with a copper anode (Tokyo, Japan). Recording conditions were angle range 3–90°, step 0.02°, recording speed 10°/min. The X-ray tube voltage was 40 kV, and the current was 15 mA. Mineral identification was performed in PDXL 2 Version 2.4.2.0 software, and the obtained diffractograms were compared with data from the ICDD PDF-2 2015 database. The detection limit of XRD analysis was about 1%.

The morphology of the concrete was analyzed with a JEOL JSM-IT300LV scanning electron microscope (SEM), operated at 20 kV (Tokyo, Japan). EDS spectra were recorded using an Oxford Instruments (Abingdon, UK) Xmax 50 mm^2^ energy dispersive X-ray spectrometer (EDS). SEM analysis was performed on sample C2/25 after 120 days of stabilization. The analysis was performed on the pre-fracture surface of concrete. The sample was subjected to XRD analysis, after which it was coated with gold (thickness 15 nm) using a JEOL JFC-1300 Auto Fine Coater for SEM-EDS analysis (Tokyo, Japan).

According to the standard SRPS EN 12457-2:2008 [37], a leaching procedure (LP) was carried out, based on which FT was characterized by compliance testing, which provides data on the leaching of granular waste materials and sludges with a liquid–solid ratio of 10 L/kg and a particle size of <4 mm. The aim of these tests is to identify heavy metals in the eluate after leaching the waste material. The tested samples were brought into contact with distilled water during continuous mixing at 10 revolutions per minute at room temperature (20 ± 2) °C, with a contact time of 24 h, without pH control. After filtration, the concentrations of the metals present (Ag, As, Ba, Be, Cd, Co, Cr, Cu, Mo, Ni, Pb, Sb, Se, Sn, V, and Zn) were determined by optical emission spectrometry of inductively coupled plasma on an ICP-OES SPECTRO CIROS Λ-125-770 nm, SPECTRO ARCOS ICP-OES analyzer, according to the standard SRPS EN ISO 11885:2007 [38]. Tests were performed in duplicate, and the results were evaluated in accordance with the relevant rulebook on categories, testing, and classification of waste, Annex 10 [39]. The TCLP test was performed to determine the toxic characteristics of tailings samples FT-7, FT-14, FT-25, and FT-40 and concrete C1/40 and C3/40 after 28 and 90 days according to US EPA standard 1311 [40]. The test material was extracted for 18 h with a slightly acidic liquid at 20 times its weight and stirred at 30 rpm at room temperature (20 ± 2) °C. After stirring, the solution was filtered, and a few drops of 1 N HNO_3_ were added. The content of heavy metals was determined using ICP-OES, according to the standard SRPS EN ISO 11885:2007 [38].

### 3.2. Preparation of Concrete Samples and Their Mix Designs

Concrete samples were made by mixing PC, aggregate, FT, and water. Portland cement brand PC 42.5N was used as a binder in the concrete. In the experiment, crushed separated natural limestone aggregate of fractions 0/4 mm, 4/8 mm and 8/16 mm was used as aggregate. Flotation tailings and pyrite concentrate, both by-products of copper ore processing at the Veliki Krivelj mine (Bor, Serbia), were used in this study. Adding pyrite concentrate to FT resulted in FT with a certain sulfur content. By replacing cement with FT, in different ratios (Table 1), and mixing with water (water/cement ratio of 0.5) according to the SRPS EN 206-1 standard [41], a concrete composite was obtained that was cast in molds of dimensions 15 × 15 × 15 cm and 10 × 10 × 10 cm. After 24 h, the hardened concrete was removed from the mold and treated under controlled conditions (temperature (20 ± 2)°C and humidity of 95%) for further testing of the concrete characteristics.

### 3.3. Flotation Tailings and Aggregate

Mixtures of FT and pyrite concentrate were prepared to achieve theoretical sulfur contents of 7%, 14%, 25%, and 40% and were denoted as FT-7, FT-14, FT-25, and FT-40, while the real concentrations of sulfur in the mixtures were 7.56, 13.84, 25.02, and 39.82%, respectively. Table 2 shows the content of oxides and total sulfur, loss on ignition in samples FT-7, FT-14, FT-25 and FT-40.

**Figure 3:**
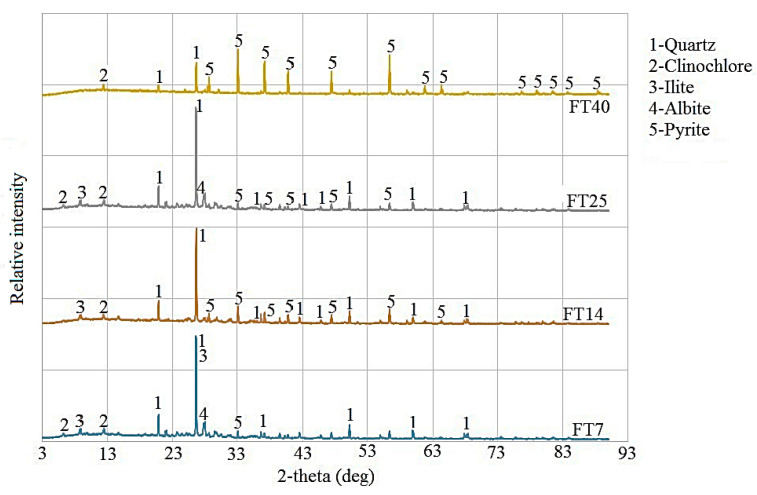
XRD analysis of flotation tailings mixtures FT-7, FT-14, FT-25, and FT-40.

The mineralogical composition of FT was determined by XRD analysis, as shown in Figure 3. Pyrite is the most abundant sulfide phase identified in all samples. Also, quartz and clinochlore are represented in all samples, while FT-7 and FT-25 additionally contain illite and albite, and FT-14 contains illite.

**Figure 4:**
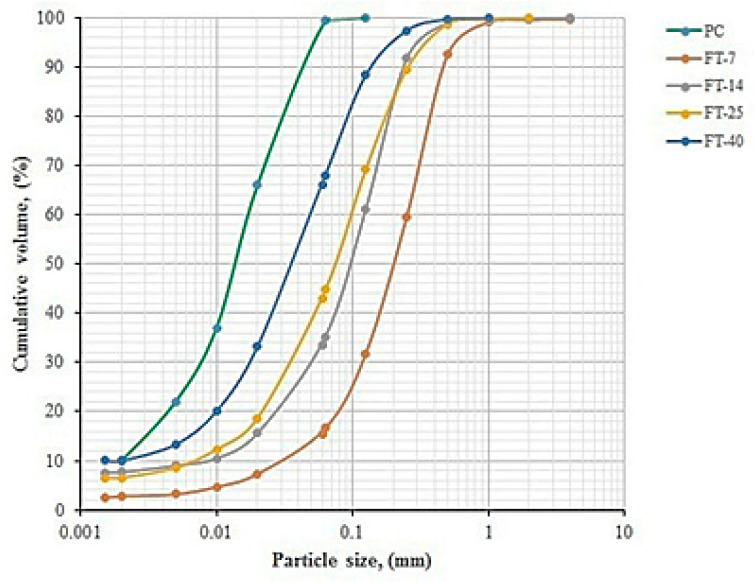
PSD of PC, FT-7, FT-14, FT-25, and FT-40.

The particle size distributions (PSDs) of flotation tailings FT-7, FT-14, FT-25, and FT-40 were determined in accordance with the SRPS EN 17892-4 standard [42]. The PSD curves are presented using a semi-logarithmic graph showing the relationship between particle size and cumulative fraction content (Figure 4). Table 3 shows the values for d_10_, d_50_, and d_90_, as well as the specific gravity (SG) of the PC, FT-7, FT-14, FT-25, and FT-40.

By comparing the d_90_ results for the samples FT-7, FT-14, FT-25, and FT-40, with values of 470.2, 240.3, 260.2, and 140.3 µm, respectively, to that of PC (d_90_ = 44.3 µm), it is evident that the FT samples contained coarser fractions than PC. Based on the PSD analysis, FT-7 had the coarsest fraction (d_90_ = 470.2 µm), which will delay the onset of the pozzolanic reaction and cause the material to behave as an inert filler during the early stages of hardening [17]. In later stages of hardening, FT-7 will exhibit chemical reactivity and initiate a pozzolanic reaction, due to its high SiO_2_ content (60.21%), as shown in Table 2 [17].

The FT-14, FT-25, and FT-40 samples will provide a physical contribution in concrete, meaning they will behave as inert fillers whose fine particles create additional nucleation sites for the hydration products—a phenomenon known as the heterogeneous nucleation effect [17].

An analysis of the d_10_, d_50_, and d_90_ values for FT-7, FT-14, FT-25, and FT-40 shows that samples with higher sulfur content (FT-25 and FT-40) exhibited a finer particle structure compared to those with lower sulfur content (FT-7 and FT-14). The fine fractions in the samples originated from sulfide minerals present in the tailings [19].

Specific gravity (SG) values range from 2.826 g/cm^3^ for low-sulfur samples (FT-7) to 4.342 g/cm^3^ in high-sulfur tailings (FT-40). This significant variation in SG is related to the variable chemical and mineralogical composition of the FT samples. Nevertheless, the measured SG values fell within the range defined by the specific gravity of quartz (SG_quartz = 2.65 g/cm^3^) and pyrite (SG_pyrite = 5.00 g/cm^3^) [19].

**Figure 5:**
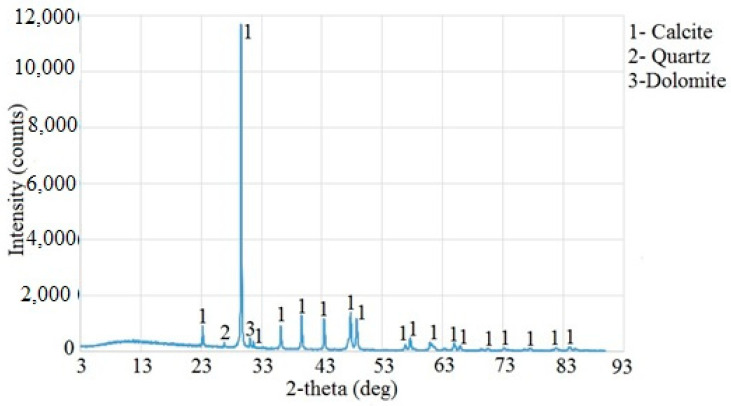
XRD analysis of limestone aggregates.

In the experiment, crushed separated natural aggregate of limestone with the fraction sizes of 0/4, 4/8, and 8/16 mm was used as an aggregate. The minerals present in the aggregate were also determined by XRD analysis (Figure 5).

### 3.4. Examination Methodology

#### 3.4.1. Assessment of the Potential of Creating Acidic Eluates

According to the standard SRPS EN 15875:2013 [43], a static test was performed to determine the acid and neutralization potential of waste containing sulfides. This standard determines the potential of sulfide-containing waste to form acidic leachate in landfills. The standard determines the method of determining the acid potential (AP) and neutralization potential (NP) of the material. From the obtained results, the net neutralization potential (NNP) was determined. AP is the maximum potential of acid formation from the sample under the assumption that all sulfur originates from pyrite and that acidity will result from its complete oxidation [43], as shown in Equation (6), where S% is the percentage of total sulfur in the sample and is expressed in kilograms of carbonate per one ton of sample, [kg CaCO_3_/t]:

AP = S% × 31.25(6)

The percentages of total sulfur in the examined tailings samples FT-7, FT-14, FT-25, and FT-40 were 7.56, 13.84, 25.02, and 39.82%, respectively. The measuring unit for AP represents the required amount of CaCO_3_ to neutralize the acidity of one ton of waste.

The neutralization potential (NP) represents the ability of the sample to neutralize the resulting acidity [43]. It is determined by adding hydrochloric acid (HCl) to the sample, then titrating it with sodium hydroxide (NaOH) to determine the amount of acid consumed by the sample. The unit of measurement for NP is expressed in kilograms of carbonate per one ton of sample, [kg CaCO_3_/t]. NP is calculated according to Equation (7), where N is the normality [mol/L], V is the volume [ml], and W is the mass of the sample [g]:

NP = [(N × V)HCl − (N × V) NaOH] × 50/W,(7)

The net neutralization potential (NNP) is calculated as the difference between NP and AP, according to Equation (8):

NNP = NP − AP(8)

If the value of NNP <−20 and NP/AP is less than 1, it indicates that the sample has the potential to generate acidic leachate because the acid potential of AP (sulfides = pyrite) is greater than the neutralization potential of NP. If the value of NNP > 20 and NP/AP is greater than 3, it means that the sample does not have the potential to generate acidic drainage water because the neutralization potential of NP (carbonates = calcite) is sufficient to neutralize the sample. If −20 < NNP < 20 and NP/AP is between 1 and 3, the acid potential is uncertain; therefore, it is necessary to perform a kinetic test [44].

#### 3.4.2. Assessment of Leaching of Heavy Metals from Monolithic Waste

In laboratory conditions, the leaching of hardened concrete was simulated to determine the amount of heavy metals in the eluate. The test was performed according to the EA NEN 7375:2004 standard [45]. After 28 days of pouring the concrete composite into 15 × 15 × 15cm molds and curing in controlled conditions (temperature (20 ± 2) °C and air humidity 95%), the concrete cubes were immersed in a container with distilled water—“tank test”. After the 0.25th, 1st, 2.25th, 4th, 9th, 16th, 36th, and 64th days after immersing the cubes, the eluate was separated, filtered, acidified, and saved for further analysis. The separated eluates, after the specified periods, were analyzed by ICP-OES SPECTRO CIROS to quantify the heavy metals prescribed by the Regulations on Categories, Testing and Classification of Waste, Annex 8, Article 2 [40]. The obtained results of the analysis were compared with the same rulebook [40].

#### 3.4.3. Compressive Strength

The compressive strength test of hardened concrete was performed according to the SRPS EN 12390-3:2019 standard [46]. With this method, the change in compressive strength during the curing period was monitored. The test was performed on the samples from Table 1 for a period of 28, 90, 120, 180, and 360 days from the day of pouring the concrete composite into molds 10 × 10 × 10 cm. The testing was done on three test bodies on the 3000 kN MATEST compression machine. After the finished care of the concrete blocks, a test must be carried out within 10 h to ensure that the sample does not lose moisture. The concrete cube is placed in the compression machine so that the force acts normal to the direction of pouring. The calculation of the compressive strength is determined according to Equation (9) [46], where fc represents the compressive strength [MPa], F is the maximum breaking load [N], and A_c_ represents the cross-sectional area of the sample affected by the compressive force [mm^2^]:

f_c_ = F/A_c_ (9)

#### 3.4.4. Concrete Porosity

The apparent porosity of permeable concrete is calculated according to Equation (10) [47]:

(10) $$p=\left[1-\frac{m_{2}-m_{1}}{\rho \times V}\right]\times 100\%$$

where p represents the apparent porosity expressed in [%], V is the volume of the dry sample [cm^3^], m_1_ is the mass of the sample measured by immersing the sample in water [g], m_2_ is the mass of the sample after 48 h immersion in water [g], and ρ is the density of water [g/cm^3^].

## 4. Results and Discussion

### 4.1. XRD Analysis

**Figure 6:**
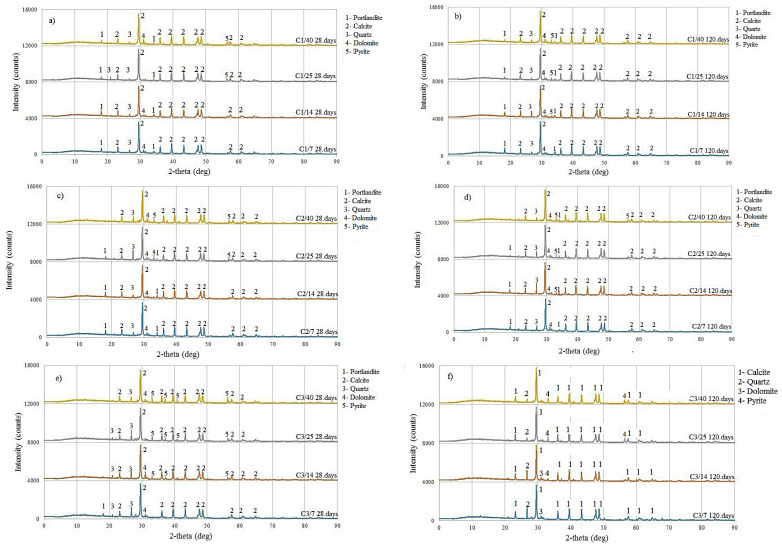
XRD analysis of samples: C1/7, C1/14, C1/25, and C1/40 after (**a**) 28 days and (**b**) 120 days; C2/7, C2/14, C2/25, and C2/40 after (**c**) 28 days and (**d**) 120 days; and C3/7, C3/14, C3/25, and C3/40 after (**e**) 28 days and (**f**) 120 days.

Semi-quantitative XRD analysis detected the minerals that make up the structure of concrete, based on which the quality and strength of concrete can be predicted. The analysis did not determine the formation of sulfate compounds gypsum, and ettringite. Figure 6 shows the comparative diffractograms of the samples from Table 1 after 28 and 120 days after casting the concrete composite into plastic molds.

In sample C1 (PC80-FT20), shown in Figure 6a,b, 20% of PC was replaced with FT. The analysis showed that portlandite and calcite, quartz, and dolomite minerals were present in all samples. Portlandite is a cement hydration product that affects the strength of concrete. According to Šestoperov (Grdić) [48], at most 10% of portlandite is formed during the hydration of cement in concrete. Calcite is the structural mineral of limestone aggregate, while quartz is present in FT. For C1/25 and C1/40, after 28 days, and C1/14, C1/25, and C1/40, after 120 days, the presence of pyrite was observed, which was expected due to the high concentration of sulfur in the concrete.

In sample C2 (PC60-FT40), shown in Figure 6c,d, 60% of PC was replaced with FT. Portlandite was detected in all samples, except for sample C2/40, which is attributed to a lower amount of cement and a higher amount of sulfide. No cement hydration products were formed in such hardened concrete [49,50,51]. A reduced cement content results in fewer hydration products, thereby decreasing compressive strength. In samples with higher concentrations of sulfur after 28 days (C2/25 and C2/40) and after 120 days (C2/14, C2/25, and C2/40), sulfide mineral pyrite was observed.

In sample C3 (PC20-FT80), shown in Figure 6e,f, 80% of PC was replaced with FT. No cement hydration products were detected in these samples, except for sample C3/7 after 28 days. This absence is attributed to the significantly reduced cement content and increased sulfide concentration, as was also observed in sample C2. The presence of the sulfide mineral pyrite was evident in all samples except C3/7 after 28 and 120 days. These samples are expected to exhibit the lowest compressive strength.

### 4.2. Scanning Electron Microscopy (SEM)

**Figure 7:**
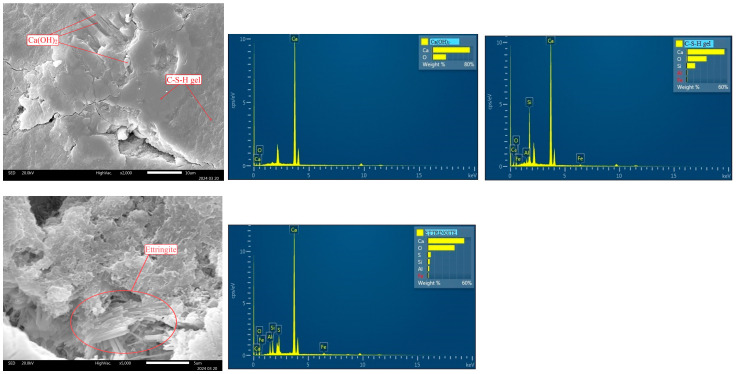
SEM images and EDS analyses of the control sample C-100.

Using SEM-EDS, the morphology and chemical composition of the control concrete sample C100 (100% cement) and the representative sample C2/25 (40% PC and 60% FT containing 25% sulfur) were analyzed after 120 days of curing. The cement hydration products tobermorite (C-S-H gel), portlandite (Ca(OH)_2_), and ettringite (3CaO·Al_2_O_3_·3CaSO_4_·32H_2_O) were detected in the sample C100 (Figure 7). Hydration products formed in the voids and pores of the concrete matrix, thereby reducing porosity and contributing to the development of a compact microstructure [5].

**Figure 8:**
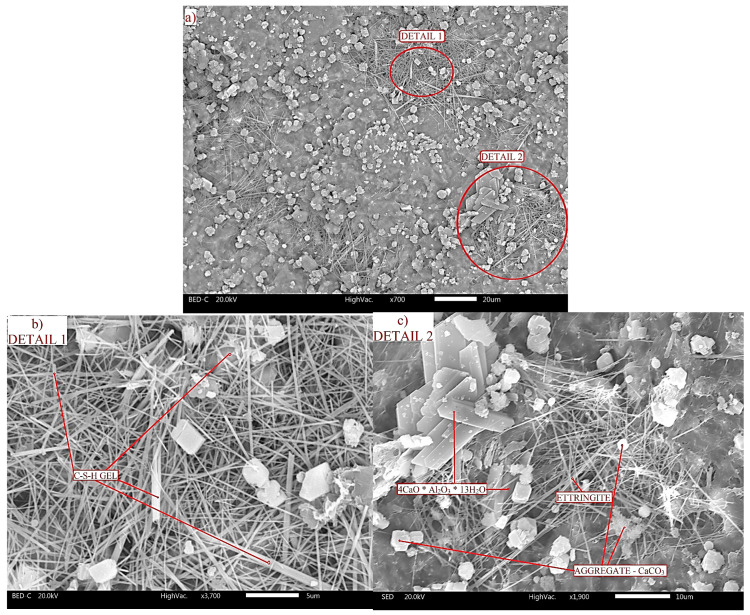
SEM image of representative sample C2/25 (**a**), with details 1 (**b**) and 2 (**c**).

The C2/25 sample was taken as a representative sample for the analysis because the analysis of the compressive strength results showed the regularity of the reduction of the compressive strength in proportion to the percentage of cement in the concrete, which is clearly expressed in the C2/14, C2/25, and C2/40 samples. Therefore, C2/25 was selected to present the morphology of the concrete and to show that the sulfur present in the concrete had no significant influence on the hydration of the cement or on the formation of new compounds (Figure 8). The obtained structure of C2/25 was homogeneous, without cracks and voids, and with the following cement hydration products: tobermorite, portlandite, ettringite, and tetracalcium aluminate hydrate (4CaO·Al_2_O_3_·13H_2_O).

Needle-like C-S-H gel [52] and plate crystals of portlandite are formed during the hydration of alite (C_3_S) and belite (C_2_S). C-S-H gel and portlandite, according to Bušatlić et al. [53], occupy about 50–60% and 20–25% of the volume of hardened hydrated cement paste, respectively. C-S-H gel is an amorphous phase [54] that plays a key role in determining the durability and mechanical properties of cementitious materials [55]. During cement hydration, C-S-H gel and portlandite fill the voids that were previously filled with water, which is why they are considered the binding phase of the cement paste. Mohamed et al. [56] explained that the fine fractions of limestone aggregate, which consists of calcite, represent nucleation points for the formation of C-S-H gel and portlandite (Ca(OH)_2_). The fine particles originating from the flotation tailings (FT) incorporated in the concrete also serve as nucleation sites for the formation of cement hydration products [17].

Ettringite is formed by hydration of tricalcium-aluminate (C_3_A) in the presence of gypsum (CaSO_4_·2H_2_O), and it consists of long needle crystals that form a network that fills the pores and cavities (Figure 8c).

Another product of hydration of tricalcium aluminate in the presence of portlandite and at a low concentration of sulfate ions (0.296 g SO_3_/1 g C_3_A) [53] is tetracalcium-aluminate hydrate (4CaO·Al_2_O_3_·13H_2_O or C_4_AH_13_; Figure 8c).This hydration reaction is the main cause of flash setting, which leads to irreversible hardening of the cement paste. The structure of the hydrate C_4_AH_13_, according to Grdić [48], consists of hexagonal plate crystals that gradually transform into the higher stable cubic form C_3_AH_6_.

The presented morphology of C2/25 indicates that the structure of the sample is homogeneous, which makes the sample slightly porous, which reduces the diffusion of oxygen and water through the concrete and prevents the oxidation of pyrite from the tailings. SEM and XRD analysis showed that no new compounds were formed during the hardening of the concrete, which indicates that there was no oxidation of pyrite in the concrete.

**Figure 9:**
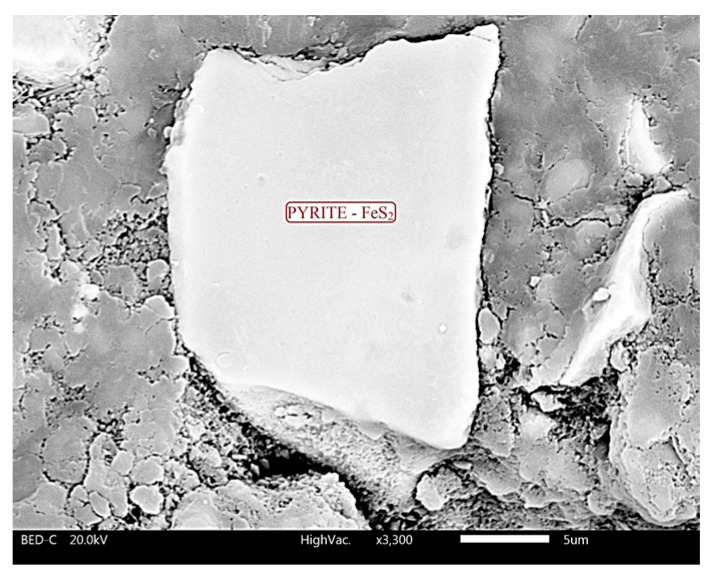
Pyrite grain in sample C2/25.

One of the catalysts of pyrite oxidation is the bacterium *Thiobacillus ferrooxidans*. Sampson et al. [57] explained that the presence of this bacterium on the pyrite surface causes mineral erosion. Figure 9 shows a pyrite grain in sample C2/25, indicating that the grain surface is undamaged, i.e., there was no mineral erosion, confirming the absence of *Thiobacillus ferrooxidans* in the concrete.

### 4.3. Uniaxial Compressive Strength

Uniaxial compressive strength tests were performed on the control sample C-100 and the samples from Table 1, C1 (PC80-FT20), C2 (PC40-FT60), and C3 (PC20-FT80), depending on the sulfur content in the flotation tailings (FT), after 28, 90, 120, 180, and 360 days of curing. The comparison of results was performed on samples with the same content of PC and FT, but with different concentrations of sulfur in the FT and curing time. This analysis aimed to determine whether the sulfur present in FT causes concrete degradation during the curing time.

Table 4 shows the measured and mean values of the uniaxial compressive strength of samples C-100, C1, C2, and C3 during the hardening period, for better visibility and easier interpretation of the obtained results. The standard deviation was determined based on the data presented in the tables, using a 95% confidence level. For statistical data analysis, Student’s t-distribution (*t*-test) was applied with degrees of freedom equal to n − 1. In our case, for five measurements, this corresponds to 4 degrees of freedom, and the corresponding t-value at the 95% confidence level was 2.7764, with the results shown in Table 3.

Table 4 presents the uniaxial compressive strength results of samples C-100, C1/7, and C1/14 after 360 days of curing, namely, 43.21, 43.91, and 44.01 [MPa], respectively. For samples C1/7 and C1/14, the compressive strength was expected to be 20% lower than that of sample C-100 (≈34.57 MPa), due to the 20% reduction in PC in samples C1/7 and C1/14. The results showed that there was no decrease in the compressive strength of the C1/7 and C1/14 samples, which means that 20% of FT-7 and FT-14 effectively replaced 20% of the PC.

The compressive strength results of samples C-100 and C2/7 after 360 days of curing were 43.21 and 27.98 [MPa], respectively. According to the proportion of PC in the sample, C2/7 had 40% PC, so the compressive strength of the C2/7 sample was expected to be 60% lower than the compressive strength of C-100. The test results showed that the compressive strength of sample C2/7 corresponded to that of a sample containing 60% PC, which means that there was a replacement of about 20% of the cement with FT-7.

As can be seen in Table 4, the compressive strengths of samples C1/25, C1/40, C2/14, C2/25, C2/40, C3/7, C3/14, C3/25, and C3/40 were lower in proportion to the reduction in the PC content in the samples. For example, C1/25 (80% PC) had a mean value of compressive strength of 30.74 MPa, and the control sample C-100 had 41.52 MPa; thus, it follows that the mean value of compressive strength of C1/25 was 25.96% lower than control sample C-100, which corresponded to a reduction of PC participation of 20% in the sample. Sample C2/25 (40% PC) had a mean compressive strength of 15.02 MPa, and control sample C-100 was 41.52 MPa, with the mean compressive strength of C2/25 being 62.83% lower than control sample C-100, which corresponded to a 60% decrease in PC participation in the sample. Sample C3/25 (20% PC) had a mean value of compressive strength of 5.92 MPa, and the control sample C-100 was 41.52 MPa, with the mean value of compressive strength of C2/25 being 85.74% less than the control sample C-100, which corresponded to an 80% decrease in PC participation in the sample.

According to the regularities shown, it can be concluded that FT-7 in samples C1 and C2, as well as FT-14 in sample C1, can replace 20% of PC. The results of previous research, ratio of mechanical results, and the number of tailings showed that FT can replace fine aggregate from 10 to 40%, and PC from 5 to 20% [58]. In order for FT to replace PC, it must have pozzolanic properties, which are proven according to the SRPS EN 450-1:2014 standard [59]. By summarizing the results of the chemical properties shown in Table 2, a comparison of the results with the requirements of the relevant standard was carried out, according to which FT-7 is classified as pozzolan of class C.

**Figure 10:**
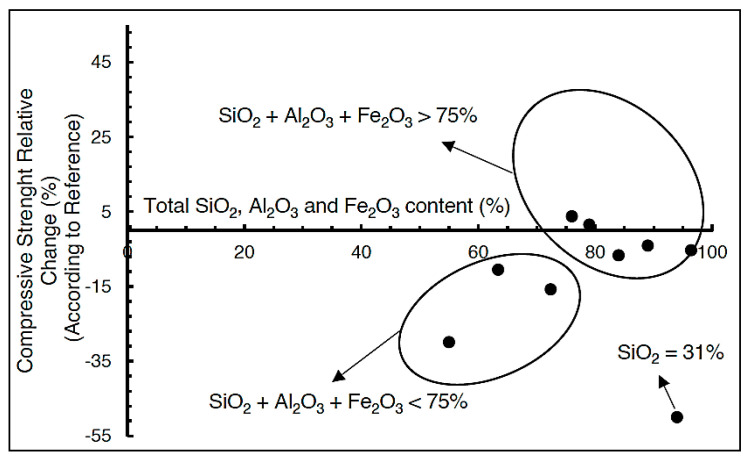
Influence of the sum of oxides SiO_2_, Al_2_O_3_, and Fe_2_O_3_ on compressive strength [59].

Due to the coarse particle content in FT-7, as shown in Table 3, in the early stages of curing, pozzolanic reactions will be delayed, and the sample will behave as an inert filler. As the curing time increases, the pozzolanic activity of FT-7 will gradually increase [17]. FT-14 does not meet the criteria of the standard, due to the high value of the loss on ignition of 12.75%, according to the standard it is max 9%, which is why it is not classified as a pozzolanic material. Deniz Adigüzel et al. [58] formed a diagram of the dependence of the relative change in compressive strength on the number of oxides in the tailings SiO_2_, Al_2_O_3_, and Fe_2_O_3_ (Figure 10), which showed that tailings with SiO_2_ + Al_2_O_3_ + Fe_2_O_3_ > 75% can have compressive strengths from 10% lower to 5% higher than the reference value.

In FT-14, the sum of SiO_2_, Al_2_O_3_, and Fe_2_O_3_ oxides was 87.68% (see Table 2), which according to the diagram means that it can be expected that the compressive strength will range from 10% lower to 5% higher than the reference sample. FT-14 had a high percentage of SiO_2_, 56.31% (Table 2), which reacts with portlandite to form calcium-silicate hydrate (C-S-H gel) responsible for the mechanical properties of concrete [58]. Sulfur, found in FT, reacts with tricalcium aluminate hydration products to form ettringite and gypsum, which lead to expansion of concrete and increase in volume, which negatively affect the mechanical properties of concrete [58]. In samples C1/7, C1/14, and C2/7, no formation of ettringite occurred, which was proven by XRD analysis (Figure 6a,b).

In samples C1, C2, and C3, the flotation tailings showed the characteristics of an inert filler in concrete. The term filler refers to particles with a maximum size of 250 µm and that they are inert, but not without influence [60,61]. Fillers can fill the intergranular space between the cement grains in concrete, thereby reducing porosity and increasing the compressive strength of concrete [62]. This is known as the fill effect. Fillers can also serve as nucleation sites for cement hydrates. This property depends on the particle size and develops in the early stage of solidification [60,61,63,64,65,66].

In samples FT-7, FT-14, FT-25, and FT-40, the contents of particles smaller than 250 µm were 59.50%, 91.70%, 89.50%, and 97.40%, respectively (see Figure 4). Samples FT-14, FT-25, and FT-40 met the conditions of the filler, due to the high content of particles smaller than 250 µm. The fine particles in FT-14, FT-25, and FT-40 samples will create spaces within the concrete where the nucleation of hydration products can occur [17].

Sulfur present in samples FT-7, FT-14, FT-25, and FT-40 in concentrations of 7.56%, 13.84%, 25.02%, and 39.82%, respectively, formed ettringite during cement hydration, which has a negative effect on the mechanical properties of concrete and leads to its degradation. In concrete samples where tailings, which contain sulfur, are used as a substitute for cement, the aggregate is natural sand, a significant decrease in compressive strength was observed with a greater participation of tailings in the sample without any clear regularity [5]. The solution to this problem is the neutralization of sulfur, which, in this paper, was achieved by using limestone as an aggregate in concrete, as will be described in detail later in the paper. By neutralizing the sulfur in the FT samples, it was found that the FT acted as a partially inert filler.

Concrete porosity is shown in Table 5 and was calculated according to Formula (10) [47] on samples C1, C2, and C3, with FT having the lowest and highest sulfur concentration.

Table 5 presents the porosity results of samples C1, C2, and C3 and the dependence of compressive strength on porosity. By comparing the porosity results of the mentioned samples with the porosity values of porous concrete, where the porosity is 15–30% [67], we can conclude that samples C1, C2, and C3 were not porous concrete. The effect of porosity on compressive strength is that with increasing porosity, compressive strength decreases, due to a decrease in the homogeneity of the sample.

The small porosity of the concrete indicates the homogeneity of the samples. Sample homogeneity is one of the basic characteristics that determine a sample’s mechanical properties, specifically compressive strength. The homogeneity of the sample was achieved by forming a sufficient amount of hydration product (C-S-H gel and portlandite), proportional participation of cement in the sample, neutralization of sulfur, use of limestone as an aggregate in the sample, and the property of FT to act as a filler in the sample due to the high percentage of fractions smaller than 250 µm.

### 4.4. Assessment of Acid Formation Potential—Static Test

In this work, a static test was performed according to SRPS EN 15875:2013 [43] on samples FT-7, FT-14, FT-25, and FT-40 in order to characterize the FT samples based on their potential for acid formation. Based on the test results (Table 6), it can be concluded that all FT samples have a potential for acid formation. The main cause for the creation of acidic drainage is the presence of pyrite in the tailings, which during oxidation, in the presence of water and oxygen, produces acid and metal ions [68]. The low neutralization potential of the samples, due to the low content of alkaline oxides K_2_O+CaO+MgO, <8 wt% [68], is also one of the reasons for the potential for acid formation.

At flotation tailings disposal sites, the potential of the tailings to form acids has a negative impact on the environment, due to the creation of acidic drainage waters. Tailings, with acid-forming potential, when used in concrete, can lead to the degradation of the concrete structure. This problem can be solved by neutralizing the sulfide in FT.

In this work, the neutralization of FT acidity was achieved by using limestone as an aggregate in concrete. The basic mineral of limestone is calcite (CaCO_3_), which is also the most commonly used mineral for neutralization [69]. A static test was performed on a representative sample C3/40 (Table 7), where the share of PC (20%), FT (80%), and sulfur concentration in FT was 39.82%, and limestone was used as aggregate. In order for the sample to meet the requirements of NAF characterization (no potential for acid formation), it is necessary that NNP > 20 and NP/AP be greater than 3 [44]. Sample C3/40 met the stated conditions, NNP = 559.23 [CaCO_3_ kg/t] and NP/AP = 4.18, which means that the neutralization of sulfur in the sample using limestone aggregate was achieved.

### 4.5. The Process of Leaching Toxic Elements

The TCLP method [17] was performed on samples of flotation tailings FT-7, FT-14, FT-25, and FT-40 and concrete C1/40 and C3/40 after 28 and 90 days in order to determine the toxic characteristics of the samples.

Table 8 presents the results of FT testing, where the concentrations of toxic elements in FT samples were lower than the threshold concentrations for non-hazardous waste according to the rulebook on categories, testing, and classification of waste [39]. Based on these results, FT is classified as non-hazardous waste.

The test results of FT-40, C1/40, and C3/40 after 28 and 90 days are shown in Table 9. C1/40 and C3/40 were taken as concrete test samples due to the higher content of sulfur in FT, which is included in the composition of concrete, in order to see the influence of sulfur and the amount of cement on the leaching of toxic elements. The amounts of tested toxic elements were lower than the permitted limits given by the rulebook [39], which classifies samples C1/40 and C3/40 as non-hazardous waste, i.e., materials that do not pose a harmful impact. It can be seen that the leaching of toxic elements was less in samples with a lower participation of FT. For example, copper leaching in concrete C1/40 after 28 days was 0.55 mg/L, and in sample C3/40, after 28 days, it was 1.11 mg/L. The reason for this is the lower participation of cement in concrete, which led to the formation of less hydration products (C-S-H gel), which resulted in greater leaching of toxic elements. The product of cement hydration, C-S-H gel, plays an important role in the stabilization of heavy metals in concrete [69]. In general, the use of flotation tailings, as a replacement for cement in concrete, contributes to the stabilization/solidification of heavy metals within the concrete matrix, thereby reducing the leaching of toxic elements into the environment. The results show that the amount of leaching of toxic elements decreased with the curing time.

### 4.6. Assessment of Leaching of Heavy Metals from Monolithic Waste

The heavy metal leaching method was performed on samples C1/6, C3/6, C1/14, C3/14, C1/25, C3/25, C1/40, and C3/40 in order to determine the amount of leached heavy metals under the influence of atmospheric conditions. In this paper, detailed test results are not presented, but only summary findings relevant to the classification of samples are given. In the second part of this paper (Part II), a complete analysis of the assessment of leaching of heavy metals from monolithic waste will be presented.

The classification and evaluation of FT pollution was conducted based on the results of leaching of heavy metals, according to the SRPS EN 12457-2 standard [37], on samples FT-6, FT-14, FT-25, and FT-40. By comparing the values, it can be seen that all the parameters met the conditions of non-hazardous waste. Samples FT-6, FT-14, FT-25, and FT-40 were classified as non-hazardous waste.

Cumulative leaching of heavy metals per unit area of the sample, as well as the rate and nature of leaching, was determined using the leaching test for monolithic waste, in accordance with the EA NEN 7375:2004 standard [45]. Based on the obtained results, the impact of samples FT-6, FT-14, FT-25, and FT-40 on the environment was determined.

The values of the measured cumulative leaching of heavy metals ($\varepsilon_{n}^{*})$ for samples C1/7, C3/7, C1/14, C3/14, C1/25, C3/25, C1/40, and C3/40 after 64 days of testing met the criteria for non-hazardous waste, which can be disposed of in landfills designated for such waste, in accordance with the rulebook [39], These findings will be discussed in greater detail in the second part of this paper (Part II).

## 5. Conclusions and Future Perspectives

Research on the application of FT as a substitute for cement in concrete structures provides a significant contribution to environmental protection, resource conservation, and the sustainable development of the construction industry. FT, which until now has been an environmental problem due to the high content of heavy metals and sulfide compounds, can be effectively converted into a valuable construction material, which achieves multiple benefits: reduction of mining waste, saving of natural raw materials/resources, and reduction of CO_2_ emissions through reduced consumption of Portland cement. This approach represents a concrete example of the implementation of the circular economy in mining and construction and can contribute to the development of sustainable cities, which is in line with the global Sustainable Development Goals (SDGs).

The results of this study indicate that the use of limestone, as a crushed separated natural aggregate, incorporated as a structural component in concrete, enabled the neutralization of sulfides originating from pyrite, which is a component of flotation tailings (FT). This conclusion is supported by the following findings:

- Acid generation potential analysis (static test) showed that the representative sample C3/40 (comprising 20% Portland cement (PC) and 80% FT, with a sulfur concentration in FT of 39.82%) has no acid generation potential (NAF). This confirms the effective neutralization of sulfides in the sample.
- Uniaxial compressive strength tests of samples C1, C2, and C3 after 360 days revealed values comparable to those recorded at 28 days. This indicates the absence of concrete degradation due to sulfide presence, further supporting the successful sulfide neutralization process.
- Microstructural and chemical analysis of sample C2/25, conducted using SEM-EDS, revealed a homogeneous structure without cracks or voids. The presence of hydration products—C-S-H gel, portlandite, ettringite, and tetracalcium aluminate hydrate—confirms that sulfides present in the concrete do not interfere with cement hydration or the formation of new compounds.
- Environmental impact assessments showed that all tested samples are environmentally benign. This was demonstrated using both the TCLP (Toxicity Characteristic Leaching Procedure) and heavy metal leaching tests on monolithic waste. TCLP analysis confirmed no leaching of toxic elements (Sb, As, Cu, Ba, Cd, Mo, Ni, Pb, Hg) from the samples. Furthermore, the leaching test for monolithic waste demonstrated that after 64 days, the eluates met the criteria for non-hazardous waste suitable for disposal at designated non-hazardous waste landfills.

However, further research is needed to optimize the relationships between flotation tailings, cement, and other components of concrete mixtures. Special emphasis should be placed on the long-term stability of concrete under conditions of different temperature and humidity regimes, as well as on extended testing of the toxicity and ecological impact of concrete with flotation tailings in real-world applications Additionally, it is recommended to explore the potential for applying this technology at a larger industrial scale, in order to reduce the environmental impact of the construction industry, while also enabling the maximum utilization of mining waste.

Future perspectives in this research area include the development of new technologies for more efficient sulfide neutralization, as well as the integration of environmental criteria in concrete production. Also, the application of flotation tailings in other industries, such as the production of cement and construction materials, could further contribute to sustainability and reduction of mining waste.

## Figures and Tables

**Table 1:** Marking of concrete samples with different proportions of PC, FT, and sulfur in FT.

Sample Abbreviation			Sample Composition
C100			100%PC
PC80-FT20 (C1)	PC80-FT20/7	C1/7	80%PC, 20%FT and 7.56% S in FT
	PC80-FT20/14	C1/14	80%PC, 20%FT and 13.84% S in FT
	PC80-FT20/25	C1/25	80%PC, 20%FT and 25.02% S in FT
	PC80-FT20/40	C1/40	80%PC, 20%FT and 39.82% S in FT
PC40-FT60 (C2)	PC40-FT60/7	C2/7	40% PC, 60% FT and 7.56% S in FT
	PC40-FT60/14	C2/14	40% PC, 60% FT and 13.84% S in FT
	PC40-FT60/25	C2/25	40% PC, 60% FT and 25.02% S in FT
	PC40-FT60/40	C2/40	40% PC, 60% FT and 39.82% S in FT
PC20-FT80 (C3)	PC20-FT80/7	C3/7	20% PC, 80% FT and 7.56% S in FT
	PC20-FT80/14	C3/14	20% PC, 80% FT and 13.84% S in FT
	PC20-FT80/25	C3/25	20% PC, 80% FT and 25.02% S in FT
	PC20-FT80/40	C3/40	20% PC, 80% FT and 39.82% S in FT

PC—Portland cement, FT—flotation tailings, S—sulfur.

**Table 2:** Chemical composition of PC, FT-7, FT-14, FT-25, and FT-40.

Component (wt.%)	PC	FT-7	FT-14	FT-25	FT-40
SiO_2_	24.56	60.21	56.31	52.14	40.21
Al_2_O_3_	4.85	17.68	14.61	8.99	4.66
Fe_2_O_3_	3.87	9.21	16.76	29.44	45.45
CaO	62.59	4.49	4.56	2.83	0.67
K_2_O	0.83	1.64	1.2	0.9	0.58
Na_2_O	0.45	1.2	1.07	0.95	0.85
MgO	2.01	1.66	0.78	0.96	0.22
P_2_O_5_	0.027	0.12	0.078	0.089	0.05
SO_3_	0.80	0.19	0.43	1.47	3.46
Cl^−^	n.d. *	n.d. *	n.d. *	n.d. *	n.d. *
Total S	0.32	7.56	13.84	25.02	39.82
Loss on ignition	-	7.99	12.75	17.29	25.71

* n.d.—not detected.

**Table 3:** Physical properties of PC, FT-7, FT-14, FT-25, and FT-40.

Sample Label	d_10_ (µm)	d_50_ (µm)	d_90_ (µm)	SG (g/cm^3^)
**PC**	2.01	14.1	44.3	3.050
**FT-7**	30.1	205.2	470.2	2.826
**FT-14**	9.1	98.2	240.3	2.962
**FT-25**	7.2	75.3	260.2	3.463
**FT-40**	2.2	36.3	140.3	4.342

d—diameter (the equivalent particle diameter).

**Table 4:** Measured and mean values of uniaxial compressive strength of control sample C-100 and samples C1, C2, and C3 during the period of curing.

**FT with Different Sulfur Content**	**C1 (PC80-FT20)**						
	**Uniaxial Compressive Strength, [MPa]**					**Mean Value of Uniaxial Compressive Strength, [MPa]**	**StDEV**
	**28 Day**	**90 Day**	**120 Day**	**180 Day**	**360 Day**		
C-100	38.97	40.68	41.88	42.87	43.21	41.52	2.151
C1/7	42.33	42.44	43.08	43.57	43.91	43.07	0.855
C1/14	42.15	42.36	43.68	43.91	44.01	43.22	1.109
C1/25	29.88	30.61	30.85	31.02	31.35	30.74	0.685
C1/40	28.38	28.74	29.95	31.39	31.97	30.09	1.961
**FT with Different Sulfur Content**	**C2 (PC40-FT60)**						
	**Uniaxial Compressive Strength, [MPa]**					**Mean Value of Uniaxial Compressive Strength, [MPa]**	**StDEV**
	**28 Day**	**90 Day**	**120 Day**	**180 Day**	**360 Day**		
C-100	38.97	40.68	41.88	42.87	43.21	41.52	2.151
C2/7	23.02	24.45	27.04	27.83	27.98	26.06	2.748
C2/14	12.49	14.32	14.9	16.66	16.97	15.07	2.270
C2/25	12.58	14.52	15.98	15.98	16.02	15.02	1.866
C2/40	14.09	15.54	16.42	16.55	16.91	15.90	1.404
**FT with Different Sulfur Content**	**C3 (PC20-FT80)**						
	**Uniaxial Compressive Strength, [MPa]**					**Mean Value of Uniaxial Compressive Strength, [MPa]**	**StDEV**
	**28 Day**	**90 Day**	**120 Day**	**180 Day**	**360 Day**		
C-100	38.97	40.68	41.88	42.87	43.21	41.52	2.151
C3/7	3.30	4.40	6.46	7.48	7.89	5.91	2.465
C3/14	3.45	5.11	6.74	7.22	7.92	6.09	2.235
C3/25	3.15	4.91	6.61	7.16	7.93	5.95	2.382
C3/40	4.01	6.03	6.23	6.60	7.77	6.13	1.691

Yellow: Control sample; Green: samples where 20% of PC is replaced by FT (FT with pozzolanic properties); Pink: samples where the compressive strength is proportional to the learning PC (FT with filler properties).

**Table 5:** Comparative table of porosity and uniaxial compressive strength of samples C1, C2, and C3.

Sample Code		Porosity [%]	Porosity StDEV	Mean Value of Uniaxial Compressive Strength [MPa]	StDEV
C1 (PC80-FT20)	T-7	3.24	1.331	43.07	2.151
	T-40	6.61	1.530	30.09	1.961
C2 (PC40-FT60)	T-7	6.82	1.594	26.06	2.748
	T-40	8.76	1.004	15.90	1.404
C3 (PC20-FT80)	T-7	9.08	1.783	5.91	2.151
	T-40	9.56	1.706	6.13	1.691

**Table 6:** Results of the static test and characterization of the FT samples.

Static Test	FT-7	FT-14	FT-25	FT-40
S, [%]	7.56	13.84	25.02	39.82
AP, [CaCO_3_ kg/t]	236.25	432.50	781.88	1244.38
NP, [CaCO_3_ kg/t]	13.13	5.25	4.25	11.75
NNP, [CaCO_3_ kg/t]	−223.13	−427.25	−777.63	−1256.13
NP/AP	0.06	0.01	0.01	−0.01
K_2_O+CaO+MgO, [%]	7.79	6.54	4.69	1.47
Sample characterization	PAF *	PAF *	PAF *	PAF *

AP—acidity potential, NP—neutralization potential, NNP—net neutralization potential. * PAF—potentially acid-forming.

**Table 7:** Results of static test C3/40.

Sample	S, [%]	AP, [CaCO_3_ kg/t]	NP, [CaCO_3_ kg/t]	NNP, [CaCO_3_ kg/t]	NP/AP	Sample Characterization
C3/40	5.62	175.63	734.85	559.23	4.18	NAF *

* NAF—not acid-forming.

**Table 8:** Chemical analysis of the eluate obtained by the TCLP method on samples FT-7, FT-14, FT-25, and FT-40.

Parameter	Unit	FT-7	FT-14	FT-25	FT-40	Reference Value for Non-Hazardous Waste *
Sb	mg/L	<0.006	<0.006	<0.006	<0.006	15
As	mg/L	<0.020	<0.020	<0.020	<0.020	5
Cu	mg/L	4.9	16.9	2.9	1.2	25
Ba	mg/L	0.080	0.063	0.061	0.036	100
Cd	mg/L	0.004	0.041	<0.004	0.007	1
Mo	mg/L	<0.007	<0.007	<0.007	<0.007	350
Ni	mg/L	0.024	0.030	0.008	<0.007	20
Pb	mg/L	0.27	0.15	0.080	1.0	5
Se	mg/L	<0.004	<0.004	<0.004	<0.004	1
Cr	mg/L	0.045	0.034	0.010	0.015	5
Zn	mg/L	2.3	2.0	0.73	0.51	250
Hg	mg/L	˂0.0005	˂0.0005	˂0.0005	˂0.0005	0.2
V	mg/L	<0.007	<0.007	<0.007	<0.007	24
Ag	mg/L	<0.005	<0.005	<0.005	<0.005	5

* Annex 10 of the rulebook on categories, testing, and classification of waste (Sl gl RS 56/2010, 93/2019, 39/2021, 65/2024), Article 1, Parameters for testing the toxic characteristics of waste intended for disposal [39].

**Table 9:** Chemical analysis of the eluate obtained by the TCLP method on samples FT-40, C1/40, and C3/40 after 28 and 90 days.

Parameter	Unit	FT-40	C1/40 after 28 Days	C3/40 after 28 Days	C1/40 after 90 Days	C3/40 after 90 Days	Reference Value for Non-Hazardous Waste *
Sb	mg/L	<0.006	0.003	0.005	0.001	0.004	15
As	mg/L	<0.020	0.0092	0.013	0.0088	0.010	5
Cu	mg/L	1.2	0.55	1.11	0.45	0.96	25
Ba	mg/L	0.036	0.021	0.033	0.018	0.03	100
Cd	mg/L	0.007	0.003	0.006	0.002	0.005	1
Mo	mg/L	<0.007	0.003	0.005	0.001	0.005	350
Ni	mg/L	<0.007	0.004	0.006	0.002	0.004	20
Pb	mg/L	1.0	0.23	0.88	0.12	0.64	5
Se	mg/L	<0.004	0.002	0.003	0.001	0.002	1
Cr	mg/L	0.015	0.0082	0.011	0.0061	0.0098	5
Zn	mg/L	0.51	0.25	0.43	0.11	0.31	250
Hg	mg/L	˂0.0005	0.0002	0.0004	0.0001	0.0003	0.2
V	mg/L	<0.007	0.002	0.006	0.001	0.004	24
Ag	mg/L	<0.005	0.002	0.004	0.001	0.003	5

* Annex 10 of the rulebook on categories, testing, and classification of waste (Sl gl RS 56/2010, 93/2019, 39/2021, 65/2024), Article 1, Parameters for testing the toxic characteristics of waste intended for disposal [39].

## Data Availability

The data presented in this study are available on request from the corresponding author due to all data will be available in the future doctoral thesis defended by Vanja Đurđevac.
